# Evaluation of recombinant monoclonal antibody SVmab1 binding to Na _V_1.7 target sequences and block of human Na _V_1.7 currents

**DOI:** 10.12688/f1000research.9918.1

**Published:** 2016-11-25

**Authors:** Dong Liu, Mandy Tseng, Linda F. Epstein, Lydia Green, Brian Chan, Brian Soriano, Desiree Lim, Oscar Pan, Christopher M. Murawsky, Chadwick T. King, Bryan D. Moyer

**Affiliations:** 1 Neuroscience, Amgen Inc., Thousand Oaks, USA; 2 Amgen British Columbia, Burnaby, Canada; 3 Molecular Engineering, Amgen Inc., Cambridge, USA; 4 Discovery Attribute Sciences, Amgen Inc., Thousand Oaks, USA

**Keywords:** NaV1.7, SVmab1, ion channel, antibody, electrophysiology

## Abstract

Identification of small and large molecule pain therapeutics that target the genetically validated voltage-gated sodium channel Na _V_1.7 is a challenging endeavor under vigorous pursuit. The monoclonal antibody SVmab1 was recently published to bind the Na _V_1.7 DII voltage sensor domain and block human Na _V_1.7 sodium currents in heterologous cells. We produced purified SVmab1 protein based on publically available sequence information, and evaluated its activity in a battery of binding and functional assays. Herein, we report that our recombinant SVmAb1 does not bind peptide immunogen or purified Na _V_1.7 DII voltage sensor domain via ELISA, and does not bind Na _V_1.7 in live HEK293, U-2 OS, and CHO-K1 cells via FACS. Whole cell manual patch clamp electrophysiology protocols interrogating diverse Na _V_1.7 gating states in HEK293 cells, revealed that recombinant SVmab1 does not block Na _V_1.7 currents to an extent greater than observed with an isotype matched control antibody. Collectively, our results show that recombinant SVmab1 monoclonal antibody does not bind Na _V_1.7 target sequences or specifically inhibit Na _V_1.7 current.

## Introduction

Ion channels are attractive drug targets and small molecule therapeutic drugs to this protein family generate worldwide sales of approximately $12 billion ^1^. Despite this attraction and the demonstrated involvement of ion channel antibodies in diverse autoimmune diseases ^2^, no antibody-based ion channel therapeutic has progressed to the clinic, due to challenges in developing both optimal immunogens and robust screening processes to identify channel modulators ^3^.

The genetically validated pain target Na _V_1.7 functions as a voltage-gated sodium channel expressed in nociceptive neurons in the peripheral nervous system ^4^. Na _V_1.7 is comprised of four domains (DI-DIV), each containing six transmembrane (TMD) helices, in which TMD helices S1–S4 contain the voltage sensor region and TMD helices S5–S6 contain the pore region. Upon membrane depolarization, the voltage sensor domains, in particular the voltage sensor paddle comprised of S3, the S3–S4 loop, and S4, move outward resulting in pore opening, influx of sodium into the cell, and action potential firing ^5^. Recently, Lee *et al*. described a monoclonal antibody SVmab1 targeted to a peptide loop between DII S3-4 in the voltage sensor paddle region, which bound a Na _V_1.7 DII voltage-sensor domain protein by ELISA and blocked Na _V_1.7 function by electrophysiology ^6^. In particular, SVmab1, purified from a hybridoma, was reported to block human Na _V_1.7 currents in a use-dependent manner, in which repeated channel opening events uncovered the epitope for antibody binding in the paddle region, akin to antibody blockade of potassium channels ^6, 7^. The antigen used to generate SVmab1 was peptide VELFLADVEG, located in the DII paddle region and the sequence of this antibody was previously reported ^8^.

We generated recombinant SVmab1 (rSVmab1) protein based on the publically available sequence information and evaluated its ability to bind peptide VELFLADVEG, purified DII voltage sensor domain protein, and cells expressing Na _V_1.7, as well as block Na _V_1.7 sodium currents in heterologous cells.

## Methods

### Cloning, expression, and purification of rSVmab1 and control antibodies

The amino acid sequences for the heavy and light chains of rSVmab1 were obtained from Table 2 of a publication ^8^. The variable region heavy chain sequence corresponds to SEQ ID NO 4 and the variable region light chain sequence corresponds to SEQ ID NO 8 of this publication. Synthetic, human codon-optimized, reverse translated DNA was generated by Genewiz, and subcloned into pTT5 expression vectors (National Research Council Canada), containing murine IgG1 heavy chain or kappa light chain constant regions. The coding regions from the resulting constructs were confirmed by sequencing to match the published sequences ^8^. Plasmids were purified (Endofree Quanta Mega Kit; MDI Healthcare Services India) and re-confirmed by both sequencing and diagnostic restriction digest prior to transfection. Heavy and light chain DNA constructs for rSVmab1 were transiently co-transfected into 1.6L of HEK293 6E cells in an Erlenmeyer shake flask.

Cells were grown in Freestyle F17 media supplemented with 4mM L-glutamine, 0.1% pluronic acid and 1x antibiotic solution (Freestyle F17: Invitrogen, #12338-026; L-glutamine: Himedia, #TC243-1Kg; Antibiotic-Antimycotic: Invitrogen, #15140-062; Pluronic F-68; Invitrogen, #24040032; Tryptone N1: TekniScience Inc, #19553). Transfections were performed using polyethylenimine (PEI; Polysciences, #23967), at a DNA–PEI MAX ratio of 1:2.88. At 24 hours post-transfection, the cells were supplemented with 0.5% Tryptone. Cells were harvested after 5 days of culture and the supernatant was used for antibody purification. Conditioned media was clarified and used for affinity chromatography using a MabSelect SuRe column (GE Healthcare Life Sciences, #17-5199-01). Fractions containing antibody were pooled and further purified by ion exchange chromatography using SP-Sepharose Fast Flow resin (GE Healthcare). Protein purification and integrity were monitored throughout by SDS-PAGE using 4–12% Bis-Tris gels (Invitrogen, #NP0322), MES SDS Running Buffer (20X; Invitrogen, #NP0002), LDS sample buffer (Invitrogen, #NP0007) and stained with Simply Blue Safe (Invitrogen, #LC6065). Purified antibody was buffer exchanged via dialysis into 10mM sodium acetate (pH5.2), containing 9% sucrose and concentrated (30kD Amicon Ultra centrifugal filter unit; Millipore, #UFC801096). The concentration of the purified antibody was determined by the A280 method on a Nanodrop 2000c (Thermo Fisher Scientific). The final antibody sample was verified by analytical size exclusion chromatography-high performance liquid chromatography (SEC-HPLC) using a YMC-Pack Diol-200, 300 × 8 mm column (YMC Co. Ltd., ID: 0830002871 P/No. DL20S05-3008WT) equilibrated with 20mM sodium phosphate, 400mM sodium chloride, at a pH 7.2, maintaining a flow rate of 0.75ml/min. Finally, the rSVmab1 preparation was assayed for endotoxin levels using the Kinetic Endotoxin Assay (Charles River PTS Assay; 1.0-0.01 EU/ml Sensitivity PTS Cartridge, #PTS2001F) and flash frozen in liquid nitrogen. The isotype-matched control antibody used for electrophysiology studies was a recombinant murine IgG1/kappa monoclonal derived from an unrelated immunization campaign. The positive control mouse monoclonal antibody, used for peptide and D2S domain binding ELISAs, was generated against the DII voltage sensor peptide sequence VELFLADVEG by Abmart, which corresponds to the exact sequence used to generate SVmab1.

### Mass spectrometry

Mass analysis of non-reduced rSVmab1 was performed on an Agilent TOF 6230 Mass Spectrometer coupled with an Agilent 1260 Infinity HPLC system. HPLC Mobile phases A and B were 0.1% trifluoroacetic acid (TFA) and 90% n-propanol/0.1% TFA, respectively. The reverse-phase column was an Agilent Zorbax 300SB-C8, 3.5µm 2.1 × 50mm column (#865750-906), heated to 75°C. A 20µg aliquot of rSVmab1 was injected into the system. The sample was chromatographed at 0.2 ml/min with an 11 min gradient as follows: 20%B for 1 min; 20–70%B over 8 min; 70–100%B over 1 min; held at 100%B for 1 min. Mass spectrometer ionization and transmission settings were set as follows: Vcap, 5900V; fragmenter voltage, 460V; nebulizer gas, 25 psig; skimmer voltage, 95V; Oct RF Vpp voltage, 800V; and drying gas, 13 l/min.

### Purification of human Na _V_1.7 DII voltage sensor domain

DNA encoding human Na _V_1.7 amino acids 709–857 (GenScript; derived from sequence NM_002977.3; https://www.ncbi.nlm.nih.gov/nuccore/NM_002977.3; NCBI Nucleotide RRID: SCR_004860) was cloned N-terminal to a 6x histidine affinity tag [D2S(709-857)-His _6_] in the pFastBac vector (Thermo Fisher Scientific), and a recombinant baculovirus was generated (Bac-to-Bac; Thermo Fisher Scientific). In total, 12L of Sf9 insect cells (3 × 10 ^6^ cell/ml; Expression Systems) were infected with 5% (v/v) virus, incubated at 27°C for 48 h in spinner flasks, harvested by centrifugation and stored at -80°C until use. The remainder of the purification was conducted at 4°C. The frozen cell pellet (175 g wet weight) was resuspended in lysis buffer [25 mM Tris-HCl (pH 7.4), 200 mM NaCl (TBS), containing 1% v/v protease inhibitor cocktail (Sigma-Aldrich, Inc., #P8340)], stirred until thawed and disrupted by passing the suspension through a high pressure homogenizer at 10,000 psi (Microfluidizer M110EHI; Microfluidics, Corp.). The crude lysate was centrifuged at 10,000 × g for 15 min and the resulting supernatant collected and centrifuged at 100,000 × g for 1.5 h in a 70 Ti rotor. The supernatant was decanted and the 100,000 × g pellet was collected, resuspended in lysis buffer and homogenized prior to solubilization. N-dodecyl-β-D-maltoside (DDM; Anatrace, Inc.) was added to the resuspended membranes to a final concentration of 40 mM, incubated for 1h on a rocker, followed by centrifugation at 100,000 × g to pellet insoluble material. The DDM soluble fraction (100ml) was decanted and used for purification. Preparative chromatography steps were performed on an AKTA Purifier (GE Lifesciences, Inc.) in TBS containing 1 mM DDM, unless noted. SDS-PAGE with Coomassie Blue staining was used to monitor purification.

Analytical tryptophan fluorescence size exclusion chromatography (Trp FSEC) was used to monitor the oligomerization state of D2S(709-857)-His _6_ during purification. Trp FSEC was performed on a Superose 6 10/300 GL column (GE Healthcare Life Sciences) equilibrated with DDM buffer, using an Agilent HPLC system equipped with a fluorescence detector (272 nm excitation/327 nm emission). Absorbance at 280nm was used to determine the protein concentration of purified D2S(709-857)-His _6_. N-terminal amino acid sequencing confirmed the identity of purified D2S(709-857)-His _6_. The DDM soluble fraction was incubated with 10ml Talon Superflow resin (Clontech) for 14–16h on a rocker. The resin was collected into an XK 16 column (GE Healthcare Life Sciences) and washed with stepwise increases in imidazole concentration (10 c.v., 5mM; 10 c.v., 7.5mM; 5 c.v., 15mM; and 2 c.v., 25mM) in DDM buffer until the A280nm reached a stable minimum. Talon-bound protein was eluted with 200mM imidazole in DDM buffer. Fractions containing D2S(709-857)-His _6_ were pooled, concentrated in Ultracel-30K MWCO ultrafiltration units (Millipore Corp., Inc.) and chromatographed on a Superdex 200 10/300 column (GE Lifesciences, Inc.) to remove contaminating proteins and imidazole. The monodispersity of fractions containing D2S(709-857)-His _6_ was confirmed by Trp FSEC ^9^. Monodisperse, micellar D2S(709-857)-His _6_ migrates at an apparent MW of 70kDa, which is similar in size to DDM micelles. Thus, the detergent concentrates during ultrafiltration and cannot be separated well using size exclusion chromatography (SEC), necessitating another Talon affinity step. SEC fractions containing monodisperse D2S(709-857)-His _6_ were pooled, and incubated with 0.5ml Talon resin for 2h. The resin was collected in a 2ml gravity column, washed, and protein was eluted with 200mM imidazole in DDM buffer. The eluate was loaded into a 0.5–3ml 10K MWCO Slide-a-Lyzer cassette (Thermo Fisher Scientific) and imidazole was removed by dialysis against DDM buffer. The dialyzed D2S(709-857)-His _6_ was collected, aliquoted, and frozen at -80°C.

### Generation of Na _V_1.7 BacMam

A recombinant BacMam baculovirus expressing human Na _V_1.7 was constructed as follows. A full-length cDNA clone of human Na _V_1.7 was obtained from Origene (pCMV6-XL4-Na _V_1.7) and codon optimized using synthetic DNAs (Thermo Fisher Scientific) to produce a cDNA that was stable during DNA propagation in *E. coli* strain HB101. The resulting cDNA was cloned into pENTR-D-Topo (Thermo Fisher Scientific) and the sequence was confirmed. pENTR-D-Topo-Na _V_1.7 was used in an LR Gateway reaction with pHTBV1.1 to produce pHTBV1.1-Na _V_1.7. After DNA sequence confirmation, pHTBV1.1-Na _V_1.7 was used in a transposition reaction to generate recombinant full-length baculoviral genomic DNA carrying Na _V_1.7, with transcription driven by the immediate early promoter from cytomegalovirus (Bac-to-Bac; Thermo Fisher Scientific). Transfection into Sf9 insect cells (Expression Systems) using FuGENE HD (Roche) allowed production of replication competent baculovirus, pseudotyped with VSV-G protein. The resulting transfection supernatant (P0 virus) was amplified twice, titered by endpoint dilution, as measured by gp64 expression (Expression Systems), and used in cell based assays.

### Stable cell lines

Human Na _V_1.7 HEK293 stably transfected cells were purchased from Eurofins Pharma Bioanalytics Services US, Inc., and human Na _V_1.7 CHO-K1 stably transfected, inducible cells were purchased from Chantest.

HEK293 complete media contained D-MEM/F-12 (1X) with 10% fetal bovine serum (FBS; US origin), 1x non-essential amino acids (NEAA; 10mM, 100X), 1x penicillin-streptomycin-glutamine (100X), and 400ug/ml Geneticin® Selective Antibiotic (all Invitrogen; #11330-033, #16000-044, 11140-050, 10378–016 and 10131-027, respectively).

CHO-K1 complete media contained F12 HAM (1X; Sigma-Aldrich, #N6658) with 10% FBS (US origin; Sigma-Aldrich, #F2442), 1x L-glutamine (Sigma-Aldrich, #G7513), 0.4mg/ml Zeocin (Invitrogen, #46-0509), and 0.01mg/ml blasticidin (Gibco, #A11139-03). CHO-K1 stable cells were seeded at 8×6 ^10^ cells in 20ml media with 1ug/ml tetracycline (Sigma-Aldrich, #T7660) and 100uM sodium butyrate (Sigma-Aldrich, #303410) in a T-175 flask and incubated 18–24hr prior to FACS analysis.

### BacMam transduction

U-2 OS cells (ATCC; #HTB-96; RRID: CVCL_0042), cultured to 80% confluency, were rinsed with Ca and Mg-free DPBS (Gibco, #14190-144) and dissociated with Cell Dissociation Buffer (enzyme-free; Gibco, #13151-014) for 8–10 minutes in a 37°C incubator. Following addition of 5.0ml of complete growth medium, cells were dislodged with gentle pipetting, pelleted, and resuspended to 3×6 ^10^ cells/5ml growth medium. Cells and human Na _V_1.7 BacMam virus added at 200 MOI were combined in a T-75 flask and incubated 18-24hr prior to FACS analysis.

U-2 OS complete media contains McCoy’s 5A with 10% FBS, 1x NEAA, 1x L-glutamine (200mM, 100X) and 1x penicillin-streptomycin (10,000U/ml, 100X) (all Gibco; #16600-082, #10099-141, #11140-050, #25030-081 and #15140-122, respectively).

### Peptide binding ELISAs

The synthetic peptide VELFLADVEG (Abmart) was conjugated to maleimide-activated bovine serum albumin (BSA; Thermo Fisher Scientific, #PI-77116) through an N-terminal cysteine. The peptide was reconstituted to 10 mg/ml in DMSO and maleimide-activated BSA was made up to 10 mg/ml in dH _2_O. The BSA-conjugate was prepared by mixing 100ug of maleimide-activated BSA in 200uL PBS, 100ug synthetic peptide and 5mM TCEP (Thermo Fisher Scientific, #PI-77720), and the reaction was incubated at room temperature overnight. BSA-conjugated synthetic peptide (VELFLADVEG) was coated at 1μg/ml on a Costar 384-well medium binding plate (#3702) using 40μL/well, in 1X PBS and incubated at 37°C for 1hr. The plate was washed three times with 90μL/well 1X PBS using a Biotek plate washer (ELx 405), blocked with 1% milk/1X PBS (90μl/well), and incubated at room temperature for 30 min. Blocking buffer was aspirated and rSVmab1 or positive control mouse monoclonal antibody against the DII sensor peptide VELFLADVEG was titrated from 200nM using 40μL/well in 1X PBS/1% milk and incubated at room temperature for 1hr. Plates were washed three times with 90μL/well 1X PBS. Polyclonal goat anti-mouse Fc HRP (Jackson ImmunoResearch Labs, #115-035-164; RRID: AB_2338510) was added at 100ng/mL in 1X PBS/1% milk (40μL/well) and incubated at room temperature for 1hr. Plates were washed an additional four times and the HRP signal was detected with 1-Step TMB (40μL/well; Neogenm #308177) for 30min followed by quenching with 1N hydrochloric acid (40μL/well). Plates were read at OD450 (Thermo Multiskan Ascent).

### Soluble DIIS binding ELISAs

Purified DIIS was coated at 2μg/ml on a 96-well NiNTA plate pre-blocked by the manufacturer with bovine serum albumin (Thermo Fisher Scientific, #15442), (50μL/well), in 1X PBS/2mM n-dodecyl-β-D-maltoside (DDM) detergent (Calbiochem, 324355), and then incubated at 37°C for 1hr. Plates were washed twice with 200μL/well of 1X PBS/2mM DDM. rSVmab1 or positive control mouse monoclonal antibody against the DII sensor peptide VELFLADVEG was titrated 1:2 from 13nM in 1% milk/1X PBS/2mM DDM (50μL/well) and then incubated at room temperature for 1hr. Following two washes with 200μL/well of 1X PBS/2mM DDM, polyclonal goat anti-mouse Fc HRP (Jackson ImmunoResearch Labs, #115-035-164; RRID: AB_2338510) was added at 400ng/mL in 1% milk/1X PBS/2mM DDM (50μL/well), and incubated at room temperature for 1hr. Plates were washed an additional four times and the HRP signal was detected with 1-step TMB (50μL/well), for 30min followed by quenching with 1N hydrochloric acid (50μL/well). Plates were read at OD450 (Thermo Multiskan Ascent).

### FACS binding assays

Human Na _V_1.7 stably transfected HEK293 cells, human Na _V_1.7 stably transfected, inducible CHO-K1 cells, human Na _V_1.7 BacMam transduced U-2 OS and parental cells were treated with non-enzymatic dissociation buffer (Sigma-Aldrich, #C5914) to remove cells from the flask prior to FACS assays. In 96-well V-bottom plates (Costar, #3897), 50,000 cells/well were incubated with 33nM rSVmab1 or isotype control (R&D Systems, #MAB002; RRID: AB_357344; monoclonal mouse IgG1 isotype control) or positive control antibodies (Millipore, #MABN41; RRID: AB_10808664; monoclonal mouse anti-human Na _V_1.7 antibody ^10^) in 50ul of FACS buffer (1X PBS+2% FBS; PBS: Hyclone, #SH30256.02; FBS: Sigma-Aldrich, #F2442, 500mL), and then incubated at 4 ^°^C for 1hr. Cells were isolated by centrifugation at 2500 RPM (664xg) for 2 min, the supernatant was removed and the cells were washed twice with 200ul/well FACS buffer. Cells were resuspended in 50ul (5ug/ml) polyclonal goat-anti-mouse IgG Fc Alexa 647 (Jackson ImmunoResearch Labs, #115-605-071; RRID: AB_2338909) and 2.5ug/ml 7-aminoactinomycin D (7AAD; Sigma, #A9400) and incubated at 4 ^°^C for 15min. Cells were then washed once, resuspended in 50ul FACS buffer and read on a Becton Dickenson Accuri Flow Cytometer using the Intellicyt Hypercyt Autosampler. Single cells were gated and geometric means (GeoMean) of 7AAD-negative cells were analyzed using the Intellicyte Forecyt 3.1 software (Intellicyt; http://intellicyt.com/products/software/). A minimum of 350 live cell events were collected per well.

### Manual patch clamp electrophysiology

Human Na _V_1.7 stably transfected HEK293 cells, plated on glass coverslips (Warner Instruments, CS-8R, #64-0701) for 18–28 hr before recording, were voltage clamped using the whole cell patch clamp configuration at room temperature (21–24°C), using a MultiClamp 700B amplifier and DIGIDATA 1322A with pCLAMP 10.2 software (Molecular Devices; https://www.moleculardevices.com/systems/conventional-patch-clamp/pclamp-10-software; RRID: SCR_011323). Pipettes, pulled from borosilicate glass capillaries (World Precision Instruments), had resistances between 1.5 and 2.0MΩ. Whole cell capacitance was uncompensated and leak subtraction was not used. Currents were digitized at 50kHz and filtered (4-pole Bessel) at 10kHz using pClamp10.2. Cells were positioned directly in front of a micropipette connected to a solution exchange manifold for antibody perfusion. The external solution consisted of 140mM NaCl, 5.0mM KCl, 2.0mM CaCl2, 1.0mM MgCl2, 10mM HEPES, and 11mM glucose, with a pH 7.4 by NaOH. The internal solution consisted of 62.5mM CsCl, 75mM CsF, 2.5mM MgCl2, 5mM EGTA, and 10mM HEPES, with a pH 7.25 by CsOH. To record from closed/resting channels, cells were held at -120mV and pulsed to -10mV for 30msec at 0.1Hz. To record from partially inactivated channels, cells were held at -120mV initially and then switched to a voltage that yielded 20% channel inactivation. 30msec pulses to -10 mV were delivered every 10 sec, and peak inward currents were recorded before and after antibody addition. To record from slow inactivated Na _V_1.7 channels (P1) and following a train of depolarizing stimuli (P26), cells were voltage clamped to -110 mV for 3 sec and sodium currents were elicited by a train of 26 depolarizations of 150msec duration to -10 mV at a frequency of 5Hz. Cells were then clamped to -20mV while 500 nM rSVmab1, isotype-matched murine IgG1/kappa monoclonal antibody derived from an unrelated immunization campaign or 0.3% BSA control was added. At the 5 and 15 minute time points post-antibody addition, cells were reclamped to -110 mV for 3sec and put through the same 26 pulse voltage protocol as above. Peak inward current during the 1 ^st^ (slow inactivated) or 26 ^th^ (use-dependent) pulse to -10 mV in the presence of antibody was divided by the peak inward current evoked by the 1 ^st^ or 26 ^th^ pulse to -10 mV in the absence of antibody to determine percent inhibition. A separate use-dependent protocol was also employed that replicated conditions used by Lee *et al.* ^6^, where cells were held at -120mV and sodium currents were elicited by a train of depolarizations of 30msec duration to -10mV at a frequency of 10Hz. All testing solutions had 0.3% BSA (Sigma-Aldrich, #A2058) to prevent non-specific adhesion of proteins to tubing and recording chamber components, and solutions were perfused over cells at 1ml/min. The pore blocker tetrodotoxin (TTX; 500 nM; Alomone Labs, #T-550) was added at the end of experiments as a positive control for robust Na _V_1.7 inhibition. Data were analyzed with pCLAMP and all figures were plotted using Origin Pro8 (OriginLab Corp).

### Statistical analysis

Electrophysiology data are presented as mean ± SEM, and statistical significance was determined using two-tailed, paired or unpaired Student's *t*-test with Origin Pro 8 software, with p<0.05 denoting statistical significance.

## Results

**Figure 1.**
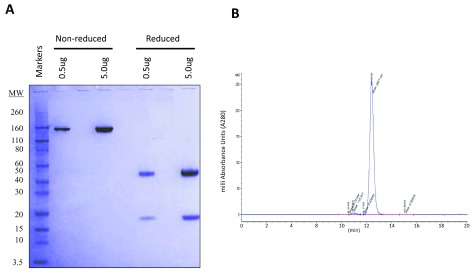
Analysis of rSVmab1. ( **A**) SDS-PAGE of 0.5 and 5.0 ug non-reduced and reduced rSVmab1. ( **B**) Size exclusion chromatography-high performance liquid chromatography elution profile of rSVmab1. The main peak comprised 97.7% of the area.

**Figure 2.**
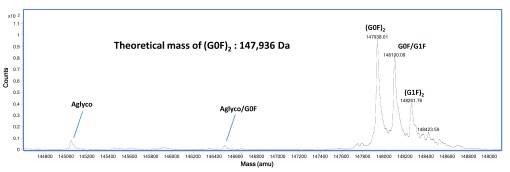
rSVmab1 evaluation by mass spectrometry. The major glycoform on non-reduced rSVmab1 is G0F (agalactosylated/fucosylated bi-antennary glycan) with a calculated mass of 147,938Da. Glycosylation of each heavy chain is denoted (G0F) _2_. Additional peaks not matching the theoretical mass of 147,936Da are extended glycoforms of the intact molecule and correspond to addition of galactoses (G1F = +1 galactose; G2F= +2 galactose) or aglyco = no glycan.

Recombinant SVmab1 (rSVmab1) was purified from transiently transfected HEK293 6E cells and analyzed by SDS-PAGE ( Figure 1A) and SEC-HPLC ( Figure 1B). rSVmab1 migrated at an observed molecular weight of ~150kDa in non-reducing SDS-PAGE, comprised distinct and appropriately sized heavy chain and light chain bands in reducing SDS-PAGE, and eluted as a single sharp peak in SEC-HPLC. Collectively, these findings are consistent with the production of an intact antibody. Mass spectrometry analysis of non-reduced rSVmab1 revealed the major peak mass to be 147,938Da, which closely matched the theoretical mass of 147,936Da for an agalactosylated/fucosylated bi-antennary glycoprotein ( Figure 2).

**Figure 3.**
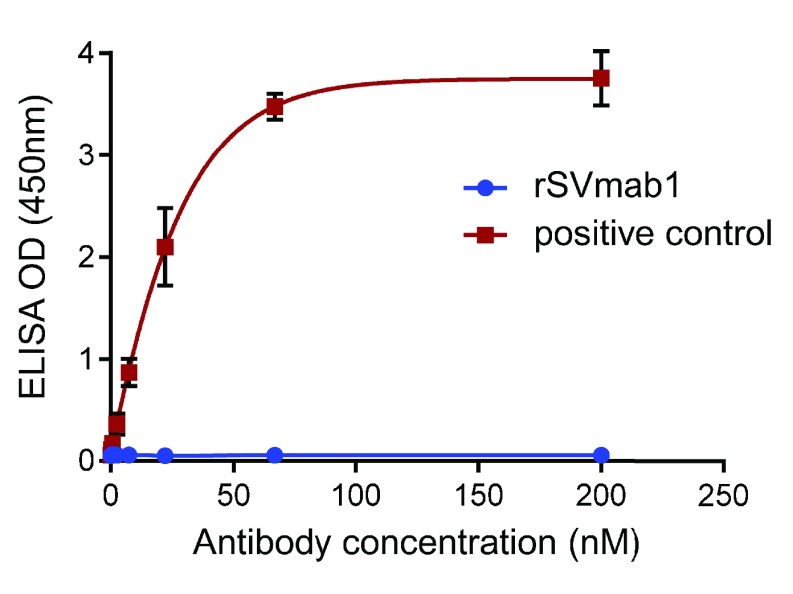
rSVmab1 does not bind to human Na _V_1.7 DII voltage sensor domain S3-S4 peptide. Peptide ELISA of increasing concentrations of rSVmab1 (blue circles) or positive control antibody (red squares) binding to the BSA-conjugated peptide VELFLADVEG. Absorbance values after subtraction of non-specific binding to uncoated plates represent means ± standard deviation of the mean of at least two independent experiments.

**Figure 4.**
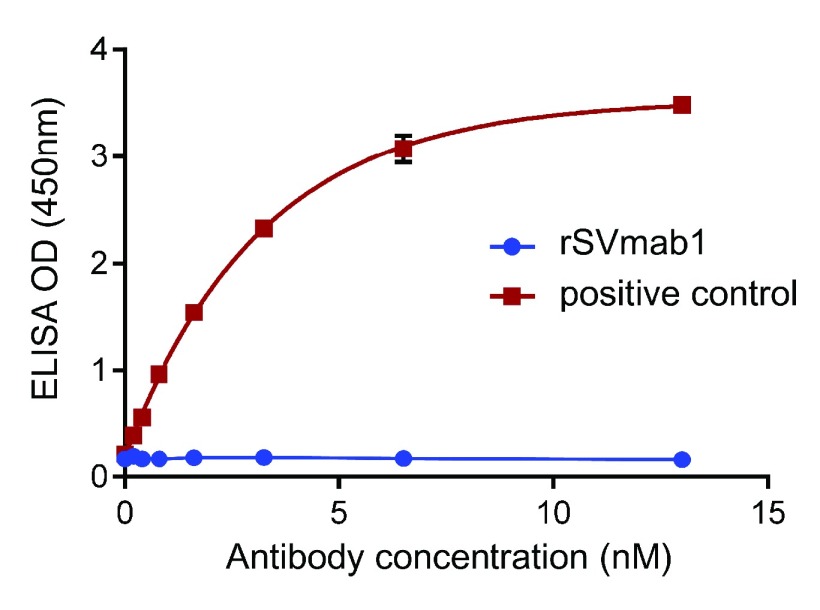
rSVmab1 does not bind to the soluble DII voltage sensor domain from human Na _V_1.7. ELISA analysis of increasing concentrations of rSVmab1 (blue circles) or positive control antibody (red squares) binding to purified, soluble Na _V_1.7 DII voltage sensor domain. Absorbance values after subtraction of non-specific binding to uncoated plates represent means ± standard deviation of the mean of at least two independent experiments.

**Figure 5.**
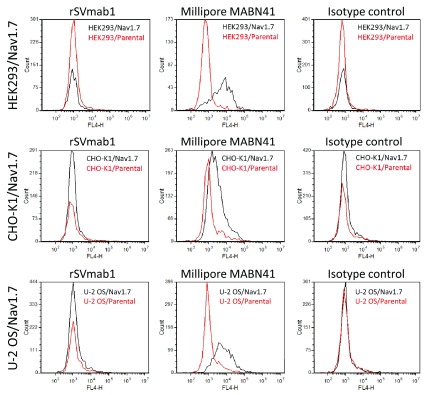
rSVmab1 does not bind to cell lines expressing human Na _V_1.7 by FACS. FACS histograms of rSVmab1, positive control Na _V_1.7 antibody (Millipore, #MABN41), and an isotype control (R&D, #MAB002) (all at 33nM) binding to cell lines stably or transiently expressing human Na _V_1.7, or their respective parental cell lines.

rSVmab1 binding to antigenic peptide was evaluated in an ELISA assay using peptide VELFLADVEG conjugated to BSA via an N-terminal cysteine residue. At 200nM rSVmab1, no peptide binding was observed, whereas binding of a positive control monoclonal antibody generated against this exact same peptide sequence was detected at a concentration as low as 2nM ( Figure 3; Dataset 1). Next, purified DII voltage sensor domain protein, housing the SVmab1 epitope, was prepared as a detergent micelle in DDM and tested for rSVmab1 binding in an ELISA assay. At 13nM rSVmab1, no DIIS binding was observed, whereas binding of the positive control antibody, described above, was detected at concentrations <1nM ( Figure 4; Dataset 2). Finally, FACS was used to assess rSVmab1 binding to HEK293, CHO-K1, and U-2 OS cells expressing human Na _V_1.7 protein. At 33nM rSVmab1, no cell binding was observed, whereas binding of a positive control Na _V_1.7 Ab was detected in all three cell lines ( Figure 5; Dataset 3).

**Figure 6.**
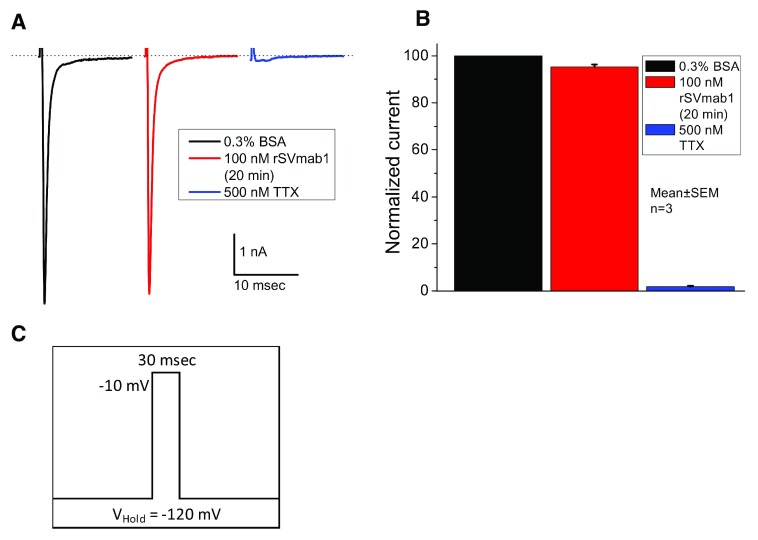
Effect of rSVmAb1 on human Na _V_1.7 channels in the resting/closed state when tested at 100 nM. ( **A**) Exemplary raw traces showing sequential addition of 0.3% bovine serum albumin (BSA) control, 100 nM rSVmab1 (after 20min incubation), and 500nM tetrodotoxin (TTX) on Na _V_1.7 currents in the same HEK293 cell. ( **B**) Summary of normalized Na _V_1.7 currents. rSVmab1 did not block Na _V_1.7 currents, whereas 500nM TTX robustly blocked Na _V_1.7 currents. Data are mean ± SEM (n=3/group). ( **C**) Voltage protocol used, where channels were held at -120 mV in the closed/resting state.

**Figure 7.**
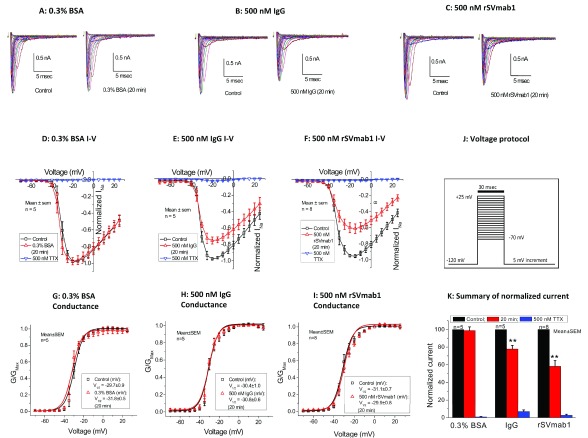
Effect of rSVmab1 on human Na _V_1.7 channels in the resting/closed state when tested at 500nM. ( **A**– **F**) Traces and I-V curves following control or 20min incubation with ( **A** and **D**) 0.3% bovine serum albumin (BSA), ( **B** and **E**) 500nM IgG, and ( **C** and **F**) 500 nM rSVmab1. ( **G**– **I**) Conductance-voltage relationships following control or 20 min incubation with ( **G**) 0.3% BSA, ( **H**) 500 nM IgG, and ( **I**) 500 nM rSVmab1. ( **J**) Voltage protocol used for panels **A**– **F**. ( **K**) Summary of normalized peak Na _V_1.7 currents from cells incubated with 0.3% BSA, 500 nM IgG, or 500 nM rSVmab1 (after 20 min incubation) followed by 500nM tetrodotoxin (TTX), which blocked nearly all current. Data are mean ± SEM (n=5–8/group). ** p<0.01 for BSA compared to IgG and BSA compared to rSVmab1 at 20 min.

**Figure 8.**
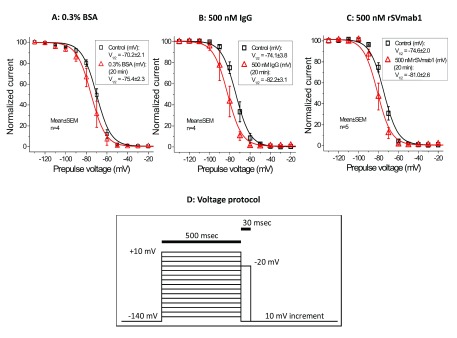
Effect of rSVmab1 on human Na _V_1.7 fast inactivation. Steady state fast inactivation curves following control or 20min incubation with ( **A**) 0.3% bovine serum albumin (BSA), ( **B**) 500nM IgG, and ( **C**) 500nM rSVmab1. Data are mean ± SEM (n=4–5/group). ( **D**) Voltage protocol used for panels **A**– **C**.

**Figure 9.**
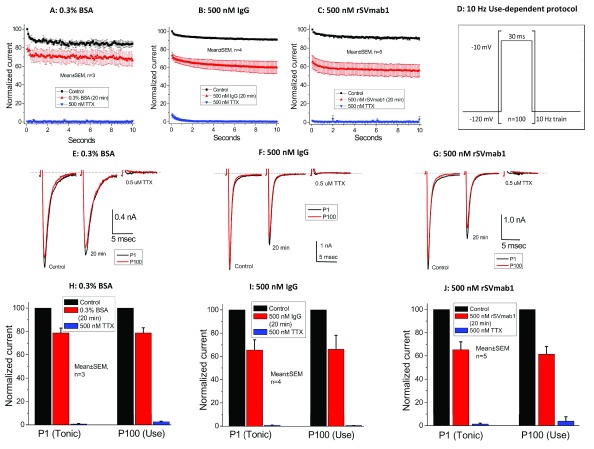
Effect of rSVmab1 on human Na _V_1.7 channels following a 10Hz use-dependent protocol. Normalized current over 100 pulses at 10Hz following control or 20min incubation with ( **A**) 0.3% bovine serum albumin (BSA), ( **B**) 500nM IgG, and ( **C**) 500nM rSVmab1. ( **D**) Voltage protocol used for panels **A**– **C**. Exemplary raw traces at pulse 1 (P1) and pulse 100 (P100) following control or 20min of incubation with ( **E**) 0.3% BSA, ( **F**) 500nM IgG, and ( **G**) 500nM rSVmab1. Summary of normalized currents at P1 (tonic block) and P100 (use-dependent block) following 20 min incubation with ( **H**) 0.3% BSA, ( **I**) 500 nM IgG, and ( **J**) 500nM rSVmab1. Data are mean ± SEM (n=3–5/group).

rSVmab1 was evaluated for functional inhibition of human Na _V_1.7 currents in HEK293 cells using whole cell manual patch clamp electrophysiology. Protocols that mimic conditions reported by Lee *et al.* ^6^, as well as protocols that interrogate diverse Na _V_1.7 gating states, were employed. Na _V_ channels exist in resting/closed states where the pore is shut, open states where sodium ions can permeate the pore, and one or more inactivated states where channels are recalcitrant to opening ^5^. When 100nM rSVmab1 was applied to cells which were voltage clamped to a holding potential of -120mV with a 0.1Hz stimulation frequency, where Na _V_1.7 channels are in the closed/resting state, no reduction of sodium current was detected following 20min of antibody treatment ( Figure 6; Dataset 4; p>0.05 comparing BSA control to rSVmab1). Notably, the pore blocker tetrodotoxin (TTX) robustly inhibited currents under these conditions. For comparison, 100 nM SVmab1 was reported to block closed/resting Na _V_1.7 by ~40% at 0.1Hz ( Figure 3D of the study by Lee *et al.* ^6^). Increasing the concentration of rSVmab1 to 500nM for 20min resulted in reductions of Na _V_1.7 current by 40% compared to reductions of 20% with an IgG1 isotype control (p=0.05 comparing rSVmab1 to IgG1 isotype control). rSVmab1 and IgG1 isotype control both yielded significantly larger current reductions compared to a BSA vehicle control group ( Figure 7; Dataset 5; p<0.01 for BSA compared to IgG1 isotype control and p<0.01 for BSA compared to rSVmab1). Conductance-voltage relationships ( Figure 7; Dataset 5) and steady-state fast inactivation curves ( Figure 8; Dataset 6) demonstrated that rSVmab1 did not affect Na _V_1.7 gating properties. rSVmab1 was next evaluated in a use-dependent protocol using a 10Hz train of depolarizing stimuli (as per Lee *et al.* ^6^) to repeatedly cycle Na _V_1.7 through open and inactive conformations in order to expose the SVmab1 epitope in the DII voltage sensor paddle region. Both 500nM rSVmab1 and an isotype control IgG1 antibody reduced tonic Na _V_1.7 current 30–35% in the first pulse of the train with nominal evidence of use-dependent block in later pulses of the train ( Figure 9; Dataset 7; p>0.05 for all group comparisons). In all these studies, antibodies were incubated on cells for 20min with constant perfusion to accommodate a potentially slow on-rate. For comparison, 100nM SVmab1 was reported to block Na _V_1.7 current over 80% within 10sec ( Figure 3C of the study by Lee *et al.* ^6^), using this 10Hz protocol.

**Figure 10.**
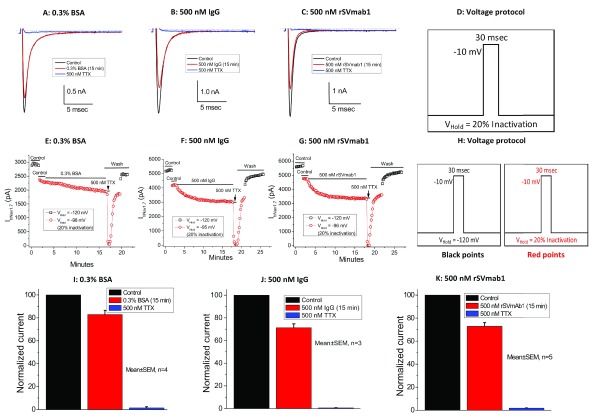
Effect of rSVmab1 on partially inactivated human Na _V_1.7 channels. Exemplary raw traces following control or 15min incubation with ( **A**) 0.3% bovine serum albumin (BSA), ( **B**) 500 nM IgG, and ( **C**) 500nM rSVmab1. ( **D**) Voltage-protocol used for panels **A**– **C**. Exemplary time courses following incubation with ( **E**) 0.3% BSA, ( **F**) 500nM IgG, and ( **G**) 500nM rSVmab1. ( **H**) Voltage protocol employed for panels **E**– **G**, where cells were held at a voltage yielding 20% channel inactivation during antibody addition. Summary of normalized currents following 15 min incubation with ( **I**) 0.3% BSA, ( **J**) 500nM IgG, and ( **K**) 500nM rSVmab1. Data are mean ±SEM (n=3–5/group).

**Figure 11.**
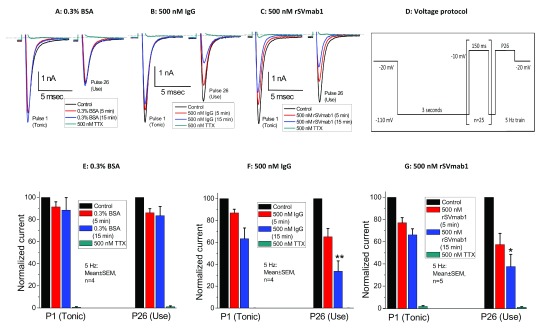
Effect of rSVmab1 on slow inactivated human Na _V_1.7 channels followed by a 5Hz use-dependent protocol. Exemplary raw traces following 5min or 15min incubation with ( **A**) 0.3% bovine serum albumin BSA, ( **B**) 500nM IgG, and ( **C**) 500nM rSVmab1. P1 = first pulse (tonic block); P26 = 26 ^th^ pulse (use-dependent block). ( **D**) Voltage protocol used for panels **A**– **C**. Cells were clamped to -20mV during addition of antibodies and between voltage measurements. Summary of normalized currents following 5 min or 15 min incubation with 0.3% ( **E**) BSA, ( **F**) 500nM IgG, and ( **G**) 500nM rSVmab1. Data are mean ± SEM (n=4–5/group). ** p<0.01 for BSA compared to IgG (15 min, P26); * p<0.05 for BSA compared to rSVmab1 (15 min, P26).

rSVmab1 was further evaluated using voltage protocols that place Na _V_1.7 channels in various inactivated states. When cells were voltage clamped at a potential that yielded 20% Na _V_1.7 inactivation, in which 20% of Na _V_1.7 channels are unavailable for opening and 80% of Na _V_1.7 channels are closed/resting, 500nM rSVmab1 and isotype control antibody decreased currents similarly around 30% after 15min of antibody treatment (p>0.05 for BSA, IgG1, and rSVmab1 comparisons), whereas TTX robustly blocked currents within seconds of application ( Figure 10; Dataset 8). When cells were evaluated using a protocol that promotes transition of Na _V_1.7 into a slow inactivated state, by maintaining cells at a resting potential of -20mV during antibody addition and between voltage measurements, 500nM rSVmab1 and isotype control IgG1 Ab both decreased currents ~35% after 15 min, whereas TTX again robustly blocked currents ( Figure 11, P1 tonic measurements; Dataset 9; p>0.05 for BSA, IgG1, and rSVmab1 group comparisons). Layering on a 5 Hz use-dependent protocol with 150msec depolarizing pulses following induction of slow inactivation resulted in current reduction by ~65% for rSVmab1 and isotype control IgG1 groups after 15min of antibody treatment ( Figure 11, P26 use measurements; Dataset 9; p<0.01 for BSA compared to IgG1, p<0.05 for BSA compared to rSVmab1, p>0.05 for IgG1 compared to rSVmab1). In these experiments, effects of rSVmab1 were similar to those of the isotype control IgG1 antibody.

## Conclusion

At the concentrations tested, recombinant monoclonal antibody SVmab1, generated from published sequence information ^8^, did not bind to the following target sources: Na _V_1.7 peptide VELFLADVEG, Na _V_1.7 DII voltage sensor protein, and Na _V_1.7 expressing mammalian cells (HEK293, CHO-K1, U-2 OS). Recombinant SVmab1 also did not specifically block Na _V_1.7 currents in HEK293 cells, as assessed by whole cell manual patch clamp electrophysiology when channels were closed/resting, inactivated, or cycled through states to expose the voltage sensor paddle region using a train of depolarizing stimuli. Reductions in Na _V_1.7 current were comparable when using an isotype control IgG1 or recombinant SVmab1 at 500nM. It is unknown why both isotype control IgG1 and recombinant SVmab1 produced current reductions larger than BSA vehicle control in some voltage protocols. In the absence of positive binding data or specific Na _V_1.7 block, our results indicate that recombinant SVmab1 is not a robust large molecule Na _V_1.7 antagonist. It should be noted that Lee *et al.* ^6^ utilized SVmab1 purified from a hybridoma, whereas the studies reported here employed recombinant SVmab1 purified from HEK293 6E cells. Differences in heavy and/or light chain antibody sequences from these sources could account for the observed differences in Na _V_1.7 binding and block. In addition, it is conceivable that differences in Na _V_1.7 glycosylation or beta subunit expression in HEK293 cells could impact epitope accessibility to SVmab1 in cell-based experiments; beta subunits have been reported to partially mask interactions between peptide toxins and Na _V_1.2 ^11, 12^. Other groups evaluating SVmab1 are encouraged to share their findings on Na _V_1.7 binding and block to inform the research community on the utility of this reagent.

## Data availability

The data referenced by this article are under copyright with the following copyright statement: Copyright: © 2016 Liu D et al.

Open Science Framework: Dataset: Evaluation of recombinant monoclonal antibody SVmab1 binding to Na _V_1.7 target sequences and block of human Na _V_1.7 currents, doi 10.17605/osf.io/4jbz7 ^13^.
