# Supplementary material for: Evaluation of recombinant monoclonal antibody SVmab1 binding to Na V1.7 target sequences and block of human Na V1.7 currents
Source: F1000Res. 2016 Nov 25;5:2764. [Version 1] doi: 10.12688/f1000research.9918.1 (PMC5155501; doi:10.12688/f1000research.9918.1)
Supplement: Peer review history

# Peer review: Referee response for version 1

**DOI:** 10.5256/f1000research.10689.r18010

**Published:** 2016-12-12

**Authors:** Theodore R. Cummins

**Affiliations:** 1 Department of Biology and Pharmacology & Toxicology, Indiana University–Purdue University Indianapolis, Indianapolis, IN, USA

This is an important study that attempts to reproduce results obtained with a putative Na _v_1.7 blocking antibody. Na _v_1.7 sodium channels have become major targets for reducing nociceptive signal transmission.  Lee *et al.* (2014) reported generation of a monoclonal antibody that binds to the S3-S4 linker of the Na _v_1.7 domain II voltage sensor, blocking channel activation and providing pain and itch relief in animals. Here Liu *et al.* used the publicly available sequence information to generate recombinant SVmab1 antibody. They rigorously characterized their antibody. Surprisingly, the antibody did not seem to bind the target immunogen, voltage-sensor or full length Na _v_1.7 channels, nor did it block Na _v_1.7 currents. This raises many important questions. The authors appropriately discuss several reasons that their results may differ from that obtained with the original SVmab1. The array of electrophysiological experiments carried out in order to detect rSVmab1 activity is impressive, but a few additions to the paper would have been helpful.

1. The lack of activity for recombinant SVmab1 raises concern about the original study and it would have been good to see if an aliquot of the original preparation from Lee *et al.* acted differently in their hands, but presumably aliquots of the original antibody are not available to the authors. Clarification of this point would have been helpful.
2. Multiple studies have identified the S3-S4 linker of the Na _v_1.7 domain II voltage sensor as critical molecular determinants of the action of peptide toxins that modulate Na _v_1.7 activity. Indeed, Huwentoxins I and IV and Protoxin II seem to target the very peptide region that SVmab1 reportedly targets ( Schmalhofer *et al.*, 2008; Sokolov *et al.*, 2008; Xiao *et al.*, 2008, 2010). Discussion of the mechanism of action of these toxins that inhibit Na _v_1.7 might have been helpful.
3. The authors generated a monoclonal antibody against the sequence putatively targeted by SVmab1. Because this control antibody did bind Na _v_1.7 in their cell lines and this peptide contains multiple major molecular determinants of HWTX-IV inhibition of Na _v_1.7 (Xiao *et al.*, 2011), it is important to know if the control antibody inhibits Na _v_1.7 currents.

Overall, this is a very interesting study. While it does not directly determine whether the voltage sensor of domain II of Na _v_1.7 is a good target for inhibiting Na _v_1.7 currents, it does raise questions about how the original SVmab1 antibody reported in Lee *et al.* (2014) was able to inhibit Na _v_1.7 currents and if binding to Na _v_1.7 domain II was indeed important for reducing pain and itch behaviors in that study.

I have read this submission. I believe that I have an appropriate level of expertise to confirm that it is of an acceptable scientific standard.

## References

[1] LeeJHParkCKChenGHanQXieRGLiuTJiRRLeeSY: A monoclonal antibody that targets a NaV1.7 channel voltage sensor for pain and itch relief. *Cell*.2014;157(6) : 10.1016/j.cell.2014.03.064 1393-404 10.1016/j.cell.2014.03.064 24856969PMC4098795

[2] SchmalhoferWACalhounJBurrowsRBaileyTKohlerMGWeinglassABKaczorowskiGJGarciaMLKoltzenburgMPriestBT: ProTx-II, a selective inhibitor of NaV1.7 sodium channels, blocks action potential propagation in nociceptors. *Mol Pharmacol*.2008;74(5) : 10.1124/mol.108.047670 1476-84 10.1124/mol.108.047670 18728100

[3] SokolovSKrausRLScheuerTCatterallWA: Inhibition of sodium channel gating by trapping the domain II voltage sensor with protoxin II. *Mol Pharmacol*.2008;73(3) : 10.1124/mol.107.041046 1020-8 10.1124/mol.107.041046 18156314

[4] XiaoYBlumenthalKJacksonJOLiangSCumminsTR: The tarantula toxins ProTx-II and huwentoxin-IV differentially interact with human Nav1.7 voltage sensors to inhibit channel activation and inactivation. *Mol Pharmacol*.2010;78(6) : 10.1124/mol.110.066332 1124-34 10.1124/mol.110.066332 20855463PMC2993464

[5] XiaoYBinghamJPZhuWMoczydlowskiELiangSCumminsTR: Tarantula huwentoxin-IV inhibits neuronal sodium channels by binding to receptor site 4 and trapping the domain ii voltage sensor in the closed configuration. *J Biol Chem*.2008;283(40) : 10.1074/jbc.M708447200 27300-13 10.1074/jbc.M708447200 18628201PMC2556013

[6] XiaoYJacksonJ.O.LiangSCumminsT.R: Common molecular determinants of tarantula huwentoxin-IV inhibition of Na channel voltage sensors in domains II and IV. *Journal of Biological Chemistry*.2011;286(31) :27301-27310 Reference source 2165952810.1074/jbc.M111.246876PMC3149324

# Peer review: Referee response for version 1

**DOI:** 10.5256/f1000research.10689.r18395

**Published:** 2016-12-12

**Authors:** Alan D. Wickenden, Wilson Edwards

**Affiliations:** 1 Molecular & Cellular Pharmacology Group, Janssen Research & Development (R&D), LLC., San Diego, CA, USA; 2 Janssen Research & Development (R&D), LLC., San Diego, CA, USA

The paper by Liu *et al.* describes the production and evaluation of a recombinant version of rSVmab1, a monoclonal antibody that was recently described to bind to the Na _v_1.7 DII voltage sensor domain, resulting in block of Na _v_1.7 sodium currents and reduction of pain and itch in rodent models (Lee *et al.,* 2014). Interesting and surprisingly,  Liu *et al*., show that recombinant SVmab1, produced with the aid of publically available sequence information, does not bind either peptide immunogen, purified Na _v_1.7 DII voltage sensor domain, or Na _v_1.7 in live HEK293, U-2 OS, and CHO-K1 cells. Consistent with the lack of binding, they further show that rSVmab has no specific effect on Na _v_1.7 currents measured in patch clamp studies employing protocols that interrogate a variety of Na _v_1.7 gating states. The study seems to have been conducted in a careful and thorough manner. In our opinion, the results conclusively show that recombinant SVmab, produced according to published sequence information, does not bind to, or inhibit Na _v_1.7, and therefore does not represent a valid tool for the exploration of Na _v_1.7 biology.

 These findings raise the important question as to why results with the recombinant form of the antibody differ so drastically from published results with SVmab purified from hybridoma. One possibility, alluded to by Liu *et al*, is that the published sequence is different from the mAb purified from the hybridoma. If the authors have access to the original hybridoma-derived SVmab, it would be interesting to see side-by-side studies with the two forms of the antibody (alternatively, it would be interesting to hear from Lee *et al*., if they already have these comparative data at hand). Although unlikely, another possibility suggested by Liu *et al*, is that differences exist in Na _v_1.7 glycosylation or beta subunit expression in the HEK293 cell lines utilized in the two studies. Since Lee *et al*., demonstrated that SVmab could block sodium currents (presumably Na _v_1.7) in small DRG neurons, it might be informative to know whether the recombinant SVmab was similarly able to block these native Na _v_1.7-mediated currents.

 The original findings by Lee *et al*, indicated that mAbs directed to the DII VSD might represent a viable strategy for inhibiting Na _v_1.7 function. Unfortunately the rSVmab described in the present study is not a useful tool for further examination of this strategy. Interestingly however, Liu *et al.*, show that the “positive control” mAb from Abmart, binds with high affinity to the peptide immunogen and the purified Na _v_1.7 DII voltage sensor domain. It would be very interesting to know if this control mAb could inhibit Na _v_1.7 function in patch-clamp studies.

 Minor points:

- rSVmab was tested at 200nM in the peptide ELISA, 13nM in the DII VSD ELISA, 33nM in the FACs assays and up to 500nM in the patch clamp studies. Why was the rSVmab tested at different concentrations in each study and why was binding not assessed at concentrations used in the functional studies?
- Page 4 & 5. Should 8x6 ^10^ and 3x6 ^10^ be 8x10 ^6^ and 3x10 ^6^?
- Why was no capacitance or series resistance compensation applied in the patch clamp studies? Please provide an estimate of typical series resistance values and associated voltage-errors.
- Please provide more information on “MABN41”. Does this mAb recognize the DII VSD or a different extracellular epitope?
- The recombinantly produced rSVmab was flash frozen following purification and prior to testing. Although many proteins tolerate freezing and thawing, including antibodies, there are some that do not, and this is a relatively minor variable worth considering. 

We have read this submission. We believe that we have an appropriate level of expertise to confirm that it is of an acceptable scientific standard.

# Peer review: Referee response for version 1

**DOI:** 10.5256/f1000research.10689.r18379

**Published:** 2016-12-09

**Authors:** David H. Hackos

**Affiliations:** 1 Department of Neuroscience, Genentech, Inc, San Francisco, CA, USA

This paper, by Dong Liu *et al*., represents a solid re-evaluation of the recombinant monoclonal antibody, named SVmab1, that was previously reported in the journal Cell (Lee *et al*., 2014) to selectively block human Nav1.7 channels in-vitro and inhibit pain behavior in-vivo.  The experiments described in this paper demonstrate that SVmab1, when expressed and purified from HEK293 cells, is not able to block Nav1.7 channels using several distinct voltage-activation protocols.  This antibody is also not able to bind to the same peptide antigen that it was reportedly raised against (Lee *et al.*, 2014), nor does it bind to the purified Nav1.7 domain II voltage-sensing domain (VSD2), nor cells expressing Nav1.7.  The experiments described in this paper were carried out with high accuracy and with the appropriate controls and described in sufficient detail in the paper.  The patch clamp analysis was especially exhaustive, testing the antibody using multiple voltage protocols covering close-state binding, partially inactivated binding, slow-inactivated binding, and use-dependent binding.  I am especially confident in these results since we have conducted a similar analysis of expressed and purified SVmab1 antibody at Genentech and also failed to detect binding or Nav1.7 blocking activity. 

 One potential concern is that the methods used to produce SVmab1 here (HEK293 cells) are different than those used by Lee *et al*., who used hybridoma expression techniques.  Perhaps there are differences in post-translational modifications between HEK293-expressed and hybridoma-expressed SVmab1, such as glycosylation, that could account for the difference in the observed effects on Nav1.7 channels.  Glycosylation differences have been previously observed when antibodies were expressed in different mammalian cell lines (Lifely *et al.*, Glycobiology. 5:813, 1995) and could in principle alter pharmacology (though probably unlikely).  Another possibility is that the sequences presented in the patent application that were used by Dong *et al.* to express SVmab1 in HEK293 cells are not correct.  This uncertainly could be resolved if the authors of Lee et al. provided detailed mass spectrometry data on their hybridoma-expressed SVmab1 and/or deposited the hybridoma to the ATCC.

 It is striking that SVmab1 lacks the ability to bind to the same peptide antigen used to generate the antibody (confirmed by Genentech).  The sequence presented in the patent application is also unusual as it lacks somatic mutations in the heavy and light variable domains (VH and VL) that are normally present in potent and specific monoclonal antibodies.  Only one non-germline residue difference is found in VL at Kabat position number 96 (W96L), which is at the VJ junction and likely due to VJ junctional diversity (not somatic mutation).  Similarly, only three non-germline residues are found in VH (in CDR 3 at Kabat positions 95, 96, and 100), which are also likely the product of junctional diversity (not somatic mutation).  Otherwise the VH segment is identical to the VhJ558.53 gene in the BALB/c mouse described by Haines et al. (Mol. Immunol. 38:9, 2001).  The lack of affinity for the peptide antigen, and the lack of somatic mutations in VH and VL, again raises the possibility that an incorrect antibody sequence is presented in the patent application.

 Minor comments:

 1.  Dong *et al*. identified a “positive control antibody” that they used as a control in binding experiments.  This antibody was generated at Abmart (which also generated SVmab1 itself) and was expressed and purified from hybridomas (instead of HEK293 cells).  However, only peptide binding and VSD2 binding data are shown (both positive).  It would be interesting to also determine whether this antibody is able to bind to native Nav1.7 (by FACS) and/or modulate Nav1.7 (by patch clamp).

 In summary, Dong *et al.* have nicely demonstrated the inability of SVmab1 to modulate Nav1.7 channels in-vitro.  These results call into question the findings of Lee *et al., *which previously had suggested the potential for identification of monoclonal antibodies capable of selectively blocking Nav1.7 both in-vitro and in-vivo.  If the antibody sequence presented in the patent application is in error or if there are essential post-translational difference between hybridoma-expressed SVmab1 and HEK293-expressed SVmab1, it is my hope that Lee *et al.* will clarify this so that their work can be appropriate replicated.

I have read this submission. I believe that I have an appropriate level of expertise to confirm that it is of an acceptable scientific standard.
